# Eucommia ulmoides seed oil is a complementary food for suppressing digestive tumors

**DOI:** 10.3389/fphar.2025.1564999

**Published:** 2025-06-18

**Authors:** Jinzheng Wu, Liang Wen, Durairaj Karthick Rajan, Yan Liu, Xin Yang, Hao Jiang, Jinhua Yan, Bo Shu, Shubing Zhang

**Affiliations:** ^1^ Department of Anesthesiology, The Second XiangYa Hospital, Central South University, Changsha, China; ^2^ Department of Cell Biology, School of Life Sciences, Central South University, Changsha, China; ^3^ Department of General Surgery, The Second Xiangya Hospital, Central South University, Changsha, China; ^4^ Department of Biomedical Informatics, School of Life Sciences, Central South University, Changsha, China; ^5^ Department of Blood Transfusion, The Third Xiangya Hospital, Central South University, Changsha, China

**Keywords:** digestive system, eucommia ulmoides seed oil, EUSO, PI3K-Akt signaling pathway, complementary food

## Abstract

**Background:**

Natural products and their bioactive components serve as valuable resources for anticancer drug discovery. Eucommia ulmoides, a medicinal and edible plant widely used in traditional medicine, contains functionally significant compounds in its seeds, particularly Eucommia ulmoides seed oil (EUSO). Previous studies have demonstrated EUSO’s promising preventive and therapeutic potential against metabolic disorders, including hypertension, diabetes, and obesity. However, its therapeutic effects on malignancies, particularly digestive system cancers, remain unexplored.

**Methods:**

To evaluate the antitumor effects of EUSO, we performed *in vitro* and *in vivo* functional analyses using Cell viability, clone formation, migration capacities, and apoptosis rates were assessed through CCK-8 assays, colony formation assays, Transwell assays, and flow cytometry in hepatocellular carcinoma (HCC) and pancreatic cancer cell models. *In vivo* antitumor efficacy was further validated using subcutaneous xenograft models in nude mice. Mechanistically, transcriptomic profiling (RNA-seq) and Western blotting were conducted to identify EUSO-regulated signaling pathways.

**Results:**

EUSO exhibited dose-dependent suppression of HCC and pancreatic cancer cell proliferation, colony formation, and migration. Flow cytometry confirmed EUSO-induced apoptosis. *In vivo*, EUSO administration suppressed tumor growth in xenograft models. Mechanistic studies revealed that EUSO downregulated PI3K-AKT-mTOR pathway activation, evidenced by reduced phosphorylation of AKT (Ser473) and mTOR (Ser2448).

**Conclusion:**

EUSO attenuates the malignant progression of digestive system cancers by inhibiting the PI3K-AKT-mTOR pathway. These results provide mechanistic evidence supporting the potential application of EUSO as an adjuvant therapeutic agent in cancer management and warrant further clinical investigation into its chemopreventive and complementary therapeutic value.

## 1 Introduction

Malignant tumors are the second most common disease affecting human health (Bray et al., 2024; Delman, 2020). Currently, standard therapeutic modalities for tumors including surgery, radiotherapy and chemotherapy. However, despite their widespread use, these approaches are limited by intrinsic challenges, including acquired drug resistance, high recurrence rates, and dose-limiting toxicities that impair patients’ quality of life (Roy and Saikia, 2016). Therefore, there is an urgent need to develop novel therapeutic agents with improved safety profiles, particularly those derived from natural products.

The PI3K-AKT signaling pathway represents a central oncogenic driver across multiple cancer types, with its hyperactivation recognized as a hallmark of malignant transformation. Mechanistically, aberrant PI3K-AKT activation accelerates tumor cell cycle progression, suppresses autophagic cell death and apoptosis. Furthermore, this pathway critically regulates epithelial-mesenchymal transition (EMT), thereby facilitating tumor invasion and metastasis. Genomic analyses have shown that genetic alterations in the PI3K-AKT-mTOR axis are present in approximately 29% of solid tumors, highlighting its clinical significance as a therapeutic target.

Targeted inhibition of PI3K/AKT signaling has emerged as a strategic approach in oncology. Natural products offer unique advantages in multi-pathway modulation, with accumulating evidence demonstrating their capacity to selectively attenuate PI3K-AKT activation. For instance, resveratrol is a natural polyphenol that has been shown to reduce bile acid-induced gastric intestinal metaplasia via the PI3K-AKT-p-FoxO4 signaling pathway (Makk-Merczel et al., 2024; Vendrely et al., 2019). Coptisine induces apoptosis in HCT-116 cells via the PI3K-AKT and mitochondria-associated apoptosis pathway (Chai et al., 2024; Yang et al., 2024), and cinnamaldehyde inhibits the progression of ovarian cancer via the PI3K-AKT pathway (Bai et al., 2023; Kim et al., 2022). These studies have shown that many natural medicines target the PI3K-AKT signaling pathway for their anti-tumor effects.

Medicinal and food homology plants, a category of natural medicines that can be used both as food and therapeutics, include examples such as ginseng and amaranth. These plants enhance the body’s resistance and immunity, effectively control inflammation, and inhibit tumor growth, occurrence, and recurrence. They are also characterized by high safety, low side effects, affordability, and high patient compliance, making them suitable for long-term use. These advantages suggest that medicinal and food homology plants have the potential to be developed into anti-tumor drugs. Eucommia ulmoides is one such medicinal and food homology plant. Its bark and leaves exhibit biological activities, including anti-tumor, antioxidant, and immunomodulatory effects. The functional oil extracted from its seeds, known as Eucommia ulmoides seed oil (EUSO), has been reported to possess anti-inflammatory, antioxidant, and lipid-lowering properties, contributing to the prevention and treatment of diseases such as hypertension, heart disease, diabetes, and obesity (Zhao et al., 2024; Yang et al., 2023; Wang et al., 2023). However, its effects on tumors remain unclear. In this study, we found that EUSO effectively inhibits cell proliferation, migration, and tumorigenesis, and induces apoptosis by suppressing the PI3K-AKT-mTOR signaling pathway. These findings provide a theoretical basis for the potential application of EUSO in adjuvant tumor therapy.

## 2 Materials and methods

### 2.1 Cell culture

Hepatocellular carcinoma cells (HepG2, Hep3B) and pancreatic cell lines (Panc-1, Miapaca-2), prostate cancer (PC-3), Non small cell lung cancer (A549), breast cancer (MDA-MB-468), and chronic myeloid leukemia (K562) were purchased from national collection of authenticated cell culture (Shanghai, China). HepG2, Hep3B Panc-1, Miapaca-2, PC-3, A549, and MDA-MB-468 cells were cultured in DMEM medium with 10% Fetal Bovine Serum (FBS), K562 was cultured in RPMI-1640 medium with 10% FBS at 37°C and 5% CO_2_ incubator.

### 2.2 CCK8 assay

Cells were diluted to 20,000 cells/mL, and 100 μL of the suspension was added to a 96-well plate. EUSO was added at various concentrations on the second day. Every 24 h(h), 100 μL of 10-fold diluted CCK8 reagent (Cat#C0005, Target Mol, USA) was added, followed by incubation for 2 h. Absorbance was measured at 450 nm using a microplate reader (Perkin Elmer, Waltham, MA, USA). Each experiment was repeated three times. The cytotoxic effect of EUSO on tumor cells and calculate by the formula: (1−OD value of EUSO-treated cells/OD value of untreated cells) × 100%.

### 2.3 Colony formation assay

Cells were diluted to 40,000 cells/mL in serum-free medium, and 100 μL of the suspension was added to a 6-well plate. EUSO was added at various concentrations on the second day. The medium was changed every 2 days, and after 1–2 weeks, cells were washed with PBS, stained with crystal violet for 30 min, and photographed for observation.

### 2.4 Transwell assay

Cells were digested with trypsin in a Petri dish, and the digestion was terminated by adding 1 mL of medium. The cells were counted under a microscope. Cells were diluted to 20,000 cells/mL in serum-free medium, and 300 μL of the suspension was added to the upper chamber. The lower chamber was added 650 μL of complete medium, and cells were treated with different EUSO concentrations. After 48 h, cells were fixed with formaldehyde for 10 min and stained with crystal violet. Photographs were taken, and the average number of cells per field was recorded.

### 2.5 Flow cytometry

2 × 10^5^ cells were seeded in a 6-well plate, and treated with EUSO at different concentrations. After 48 h, cells were collected, resuspended in 1 mL of PBS, centrifuged at 1,000 rpm for 10 min, and incubated with the Annexin V-FITC/propidium iodide (PI) apoptosis detection kit (Cat#88–8005–74, eBioscience, USA) according to the manufacturer’s protocol. Apoptosis levels were measured using flow cytometry (FACS Calibur; BD Biosciences, San Jose, CA, USA).

### 2.6 RNA-seq

Two groups of cells were treated with EUSO (experimental group) or DMSO (control group) for 48 h. The medium was discarded, and cells were washed with PBS three times. Trizol (1 mL) was added, and the cells were collected. Biomarker Technologies was commissioned to perform RNA-seq and subsequent bioinformatics analysis.

### 2.7 Western blot analysis (WB)

Cell samples from different treatments were washed twice with cold PBS (4°C). RIPA lysis buffer (100 μL) with 1% protease and phosphatase inhibitors was added to each group, and cells were lysed on ice for 5 min. The lysates were transferred to 1.5 mL tubes, incubated on ice for 30 min, and centrifuged at 12,000 rpm for 10 min at 4°C. Protein quantification, denaturation, electrophoresis, and membrane transfer were performed, followed by blocking with 5% skim milk for 2 h. Primary antibodies were incubated overnight at 4°C, followed by washing with TBST. Secondary antibodies were incubated for 2 h at room temperature, and protein bands were detected using a chemiluminescence detection system (BIO-RAD, USA). Data were quantified using Uvitec Alliance software (Eppendorf, Hamburg, Germany).

### 2.8 *In vivo* xenograft

BALB/c nude mice (5–7 weeks old) were randomly assigned to four groups. A total of 2 × 10^7^ HepG2 or Panc-1 cells were injected subcutaneously. Once tumors reached approximately 60–80 mm^3^. After the tumor model was successfully established, the nude mice were randomly divided into two groups. One group received 0.4 mL of EUSO treatment via oral gavage twice daily for 14 consecutive days, while the other group was administered the same volume of corn oil using the same regimen. After 2 weeks, the mice were euthanized, and tumor volumes (mm^3^) were measured using formula V = (W^2^ × L)/2 (L = longest diameter). Tumor tissues were preserved in formaldehyde and embedded for subsequent analysis. All animal protocols were approved by the Animal Experimental Ethical Committee, Central South University, in accordance with the guidelines for the Care and Use of Laboratory Animals. The study involving human participants was approved by the Ethical Committee of the School of Life Sciences (No. 2022–03–62), Central South University.

### 2.9 Statistical analysis

For each experiment, at least three times of repeats were performed. Data were analyzed using SPSS 25.0 statistical software. Parametric data were analyzed using two-tailed t-tests or one-way ANOVA, while non-parametric data were analyzed using the Kruskal–Wallis one-way ANOVA on ranks. Statistical significance was defined as: *p < 0.05, **p < 0.01 and ***p < 0.001.

## 3 Results

### 3.1 EUSO inhibits cell proliferation and clone formation in human digestive system cancer cells

**FIGURE 1:**
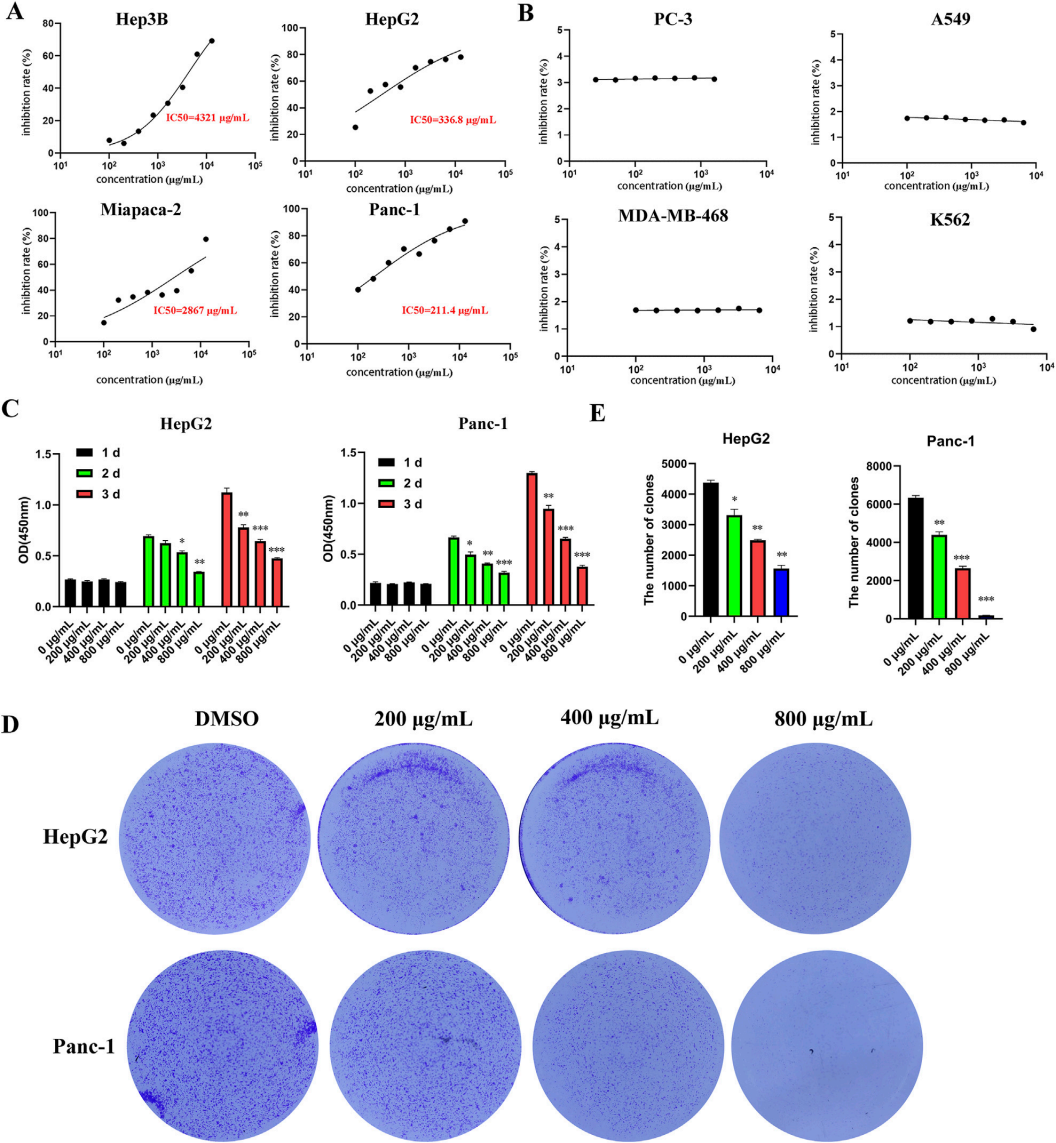
EUSO can inhibit the proliferation and clone formation of hepatocellular carcinoma and pancreatic cancer cells *in vitro* experiments. **(A)** CCK8 was used to detect the cell viability of tumor cells treated with different concentrations of EUSO for 48 h and calculate the half maximal inhibitory concentration of cells. [**(A)** Hep3B; **(B)** HepG2; **(C)** Miapaca-2; **(D)** Panc-1] in a concentration-dependent manner; Among them, the 50% inhibitory concentration of HepG2 and Panc-1 cells with high malignancy is relatively low, **(B)** EUSO had no significant effect on the proliferation of PC-3, A549, MDA-MB-468, K562, **(C)** cell viability gradually decreased with longer durations of EUSO treatment, **(D,E)** The colony forming experiment showed EUSO inhibited HepG2 and Panc-1 cell clone formation. The difference was statistically significant. **p < 0.01, ***p < 0.001.

To investigate the effect of EUSO on the viability of various tumor cells *in vitro*, different tumor cells were treated with various doses of EUSO for 2 days. The results showed that EUSO significantly inhibited the proliferation of HCC(HepG2: IC50 = 336.8 μg/mL, Hep3B: IC50 = 4.3 mg/mL) and pancreatic cancer cell lines (Panc-1: IC50 = 211.4 μg/mL, Miapaca-2: IC50 = 2.9 mg/mL) in a concentration-dependent manner (Figure 1A), which had no significant effect on the proliferation of PC-3, A549, MDA-MB-468, K562 (Figure 1B). Then, cell viability and clone formation gradually decreased with longer durations of EUSO treatment (Figures 1C–E). These results indicate that EUSO inhibits cell proliferation in human digestive system cancer cells.

### 3.2 EUSO inhibits cell migration in human digestive system cancer cells

**FIGURE 2:**
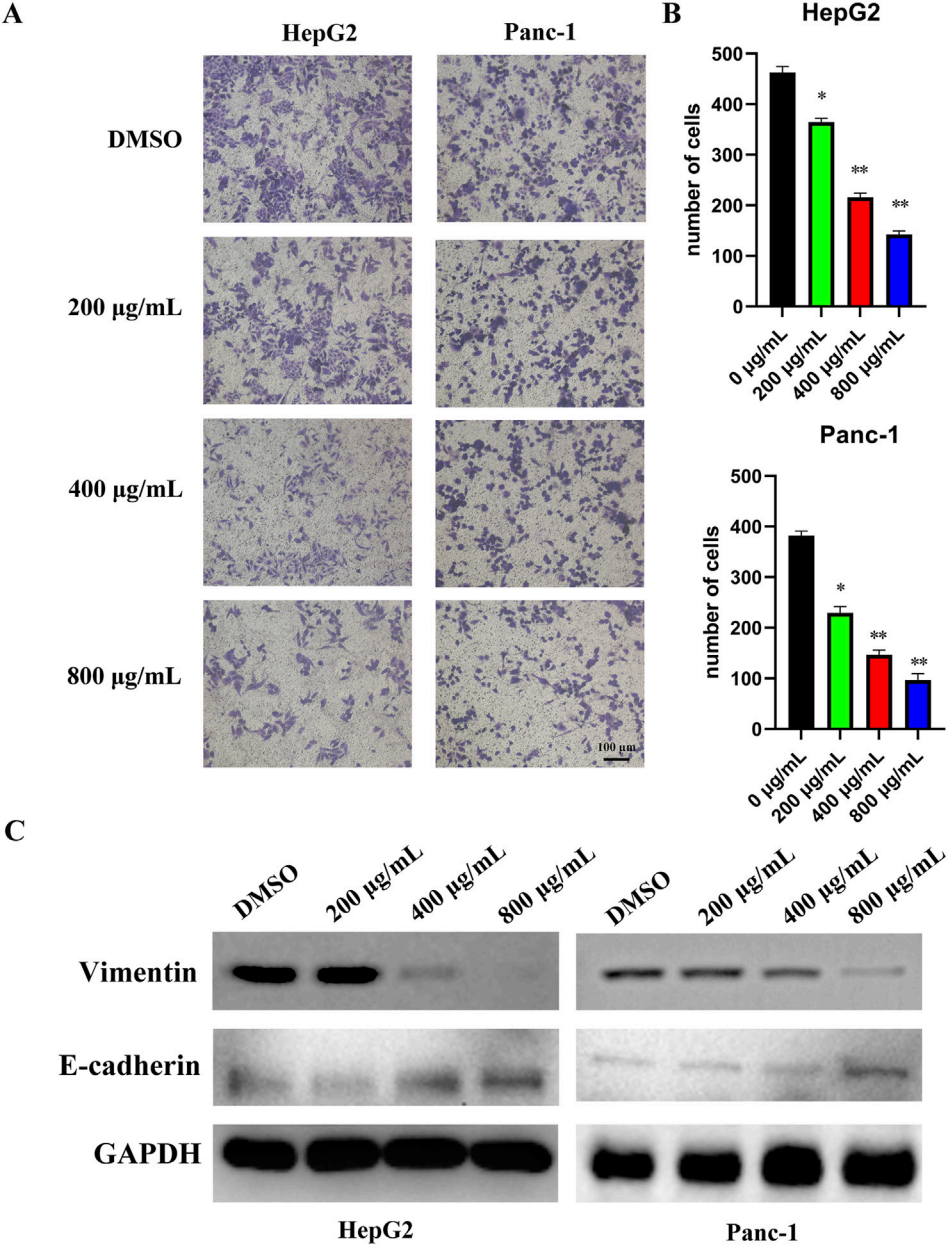
EUSO inhibits cell migration in human digestive system cancer cells. D ImageJ software was used to calculate the number of cell clone, **(A)** Transwell results revealed that EUSO inhibited HepG2 and Panc-1 migration, **(B)** ImageJ software was used to calculate the number of cell migration, **(C)** Western blot analysis further revealed that EUSO treatment decreased vimentin expression and increased E-cadherin levels, the difference was statistically significant. **p < 0.01, ***p < 0.001.

Transwell assays demonstrated that EUSO inhibited the migration of HepG2 and Panc-1 cells (Figures 2A,B). Western blot analysis further revealed that EUSO treatment decreased vimentin expression and increased E-cadherin levels (Figure 2C). Together, these findings indicate that EUSO selectively inhibits the migration of human digestive system cancer cells.

### 3.3 EUSO could induce tumor cell apoptosis by regulating the expression of cleaved caspase-3 and Bcl2

**FIGURE 3:**
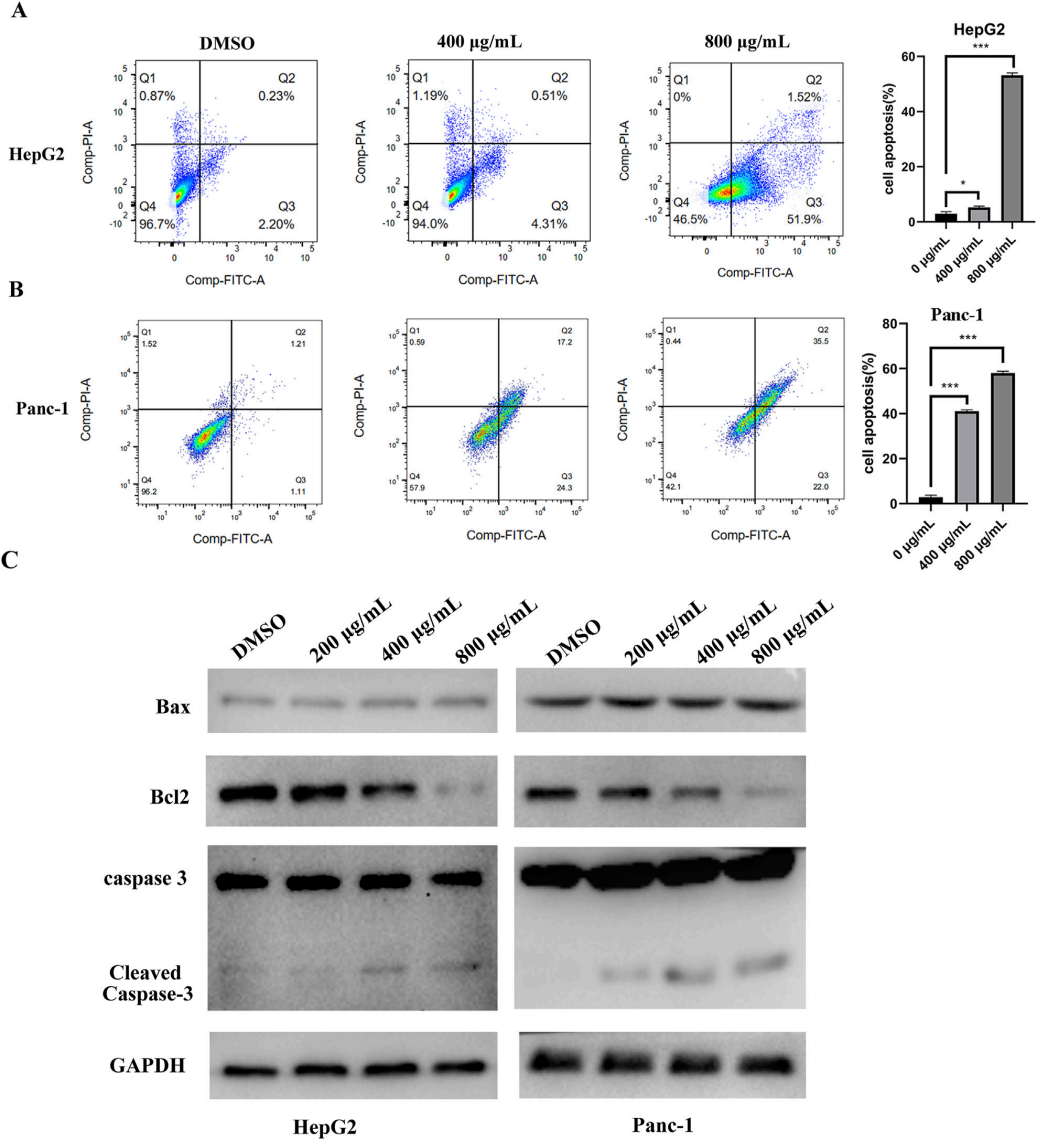
EUSO can induce cancer cells apoptosis. **(A)** The apoptosis rate of Panc-1 cells was increased after EUSO(0, 400, 800 μg/mL) treatment by flow cytometry, **(B)** The apoptosis rate of HepG2 cells was increased after EUSO(0, 400, 800 μg/mL) treatment by flow cytometry, **(C)** WB experiment showed that the expression of Bcl2 decreased and the expression level of cleaved caspase-3 increased after EUSO(0, 400, 800 μg/mL) treatment, the difference was statistically significant. **p < 0.01, ***p < 0.001.

Cell apoptosis was assessed by flow cytometry, which revealed an increased apoptosis rate in HepG2 and Panc-1 cells following EUSO treatment (Figures 3A,B). WB experiment showed that the expression of Bcl2 decreased, while the expression level of cleaved caspase-3 increased following EUSO treatment (Figure 3C). These results suggest that EUSO induces apoptosis in human digestive system cancer cells by modulating the expression of Bcl-2 and cleaved caspase-3.

### 3.4 EUSO inhibits PI3K-AKT-mTOR signaling pathway

**FIGURE 4:**
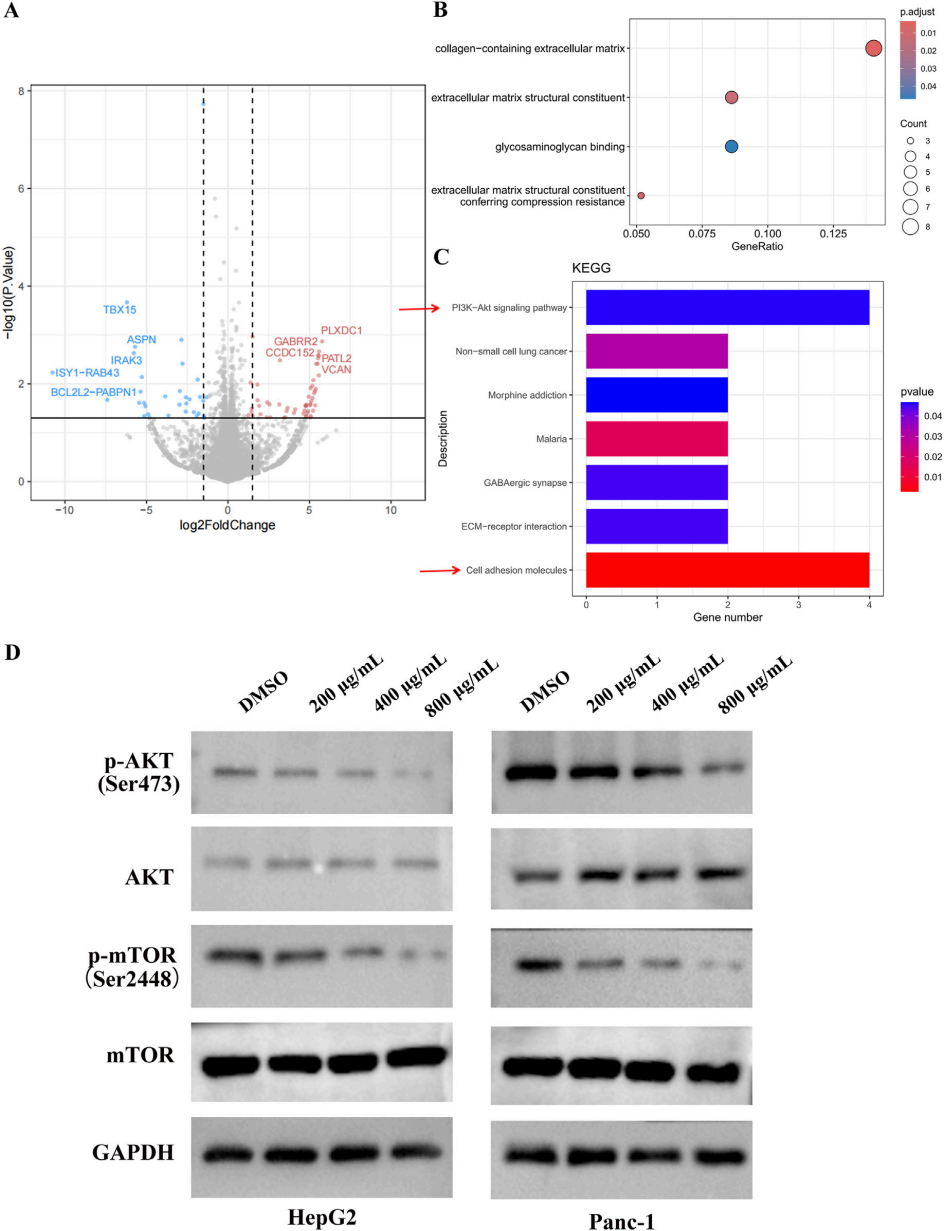
EUSO inhibits PI3K-AKT signaling pathways. **(A)** Volcano plots showed a total of 148 differentially changed genes were detected by EUSO treatment from RNA-seq, **(B)** GO analyzed found that the differential genes were enriched to: collagen-containing extracellular matrix, constituent, glycosaminoglycan binding, and extracellular matrix structural, **(C)** KEGG pathway enrichment analysis of differentially expressed genes showed that PI3K-AKT signaling pathway was significantly enriched, **(D)** E WB results showed that EUSO reduced the expression of p-AKT and p-mTOR protein in HepG2 and Panc-1, the difference was statistically significant. **p < 0.01, ***p < 0.001.

To investigate the tumor-suppressive mechanism of EUSO in cancer cells, RNA sequencing (RNA-seq) was performed to assess gene expression changes following EUSO treatment. Differential expression was visualized using volcano plots based on fold change (FC) and p-values. A total of 148 differentially expressed genes were identified, including 50 significantly downregulated genes (shown in blue) and 98 significantly upregulated genes (shown in red). The top five upregulated genes were PLXDC1, GABRR2, CCDC152, PATL2, and VCAN, while the top five downregulated genes were TBX15, ASPN, IRAK3, ISY1-RAB43, and BCL2L2-PABPN1 (Figure 4A).

Gene Ontology (GO) analysis revealed that the differentially expressed genes were significantly enriched in terms related to the collagen-containing extracellular matrix, glycosaminoglycan binding, and extracellular matrix structure (Figure 4B). KEGG pathway enrichment analysis further indicated significant enrichment in the PI3K-AKT-mTOR signaling pathway (Figure 4C). Consistently, Western blotting (WB) showed that EUSO treatment reduced the expression of phosphorylated AKT (p-AKT, Ser473) and phosphorylated mTOR (p-mTOR, Ser2448) proteins in HepG2 and Panc-1 cells (Figure 4D). Ogether, these results suggest that EUSO suppresses tumor cell function by downregulating the PI3K-AKT-mTOR signaling pathway.

### 3.5 EUSO inhibits tumor malignant transformation through the PI3K-AKT-mTOR pathway

**FIGURE 5:**
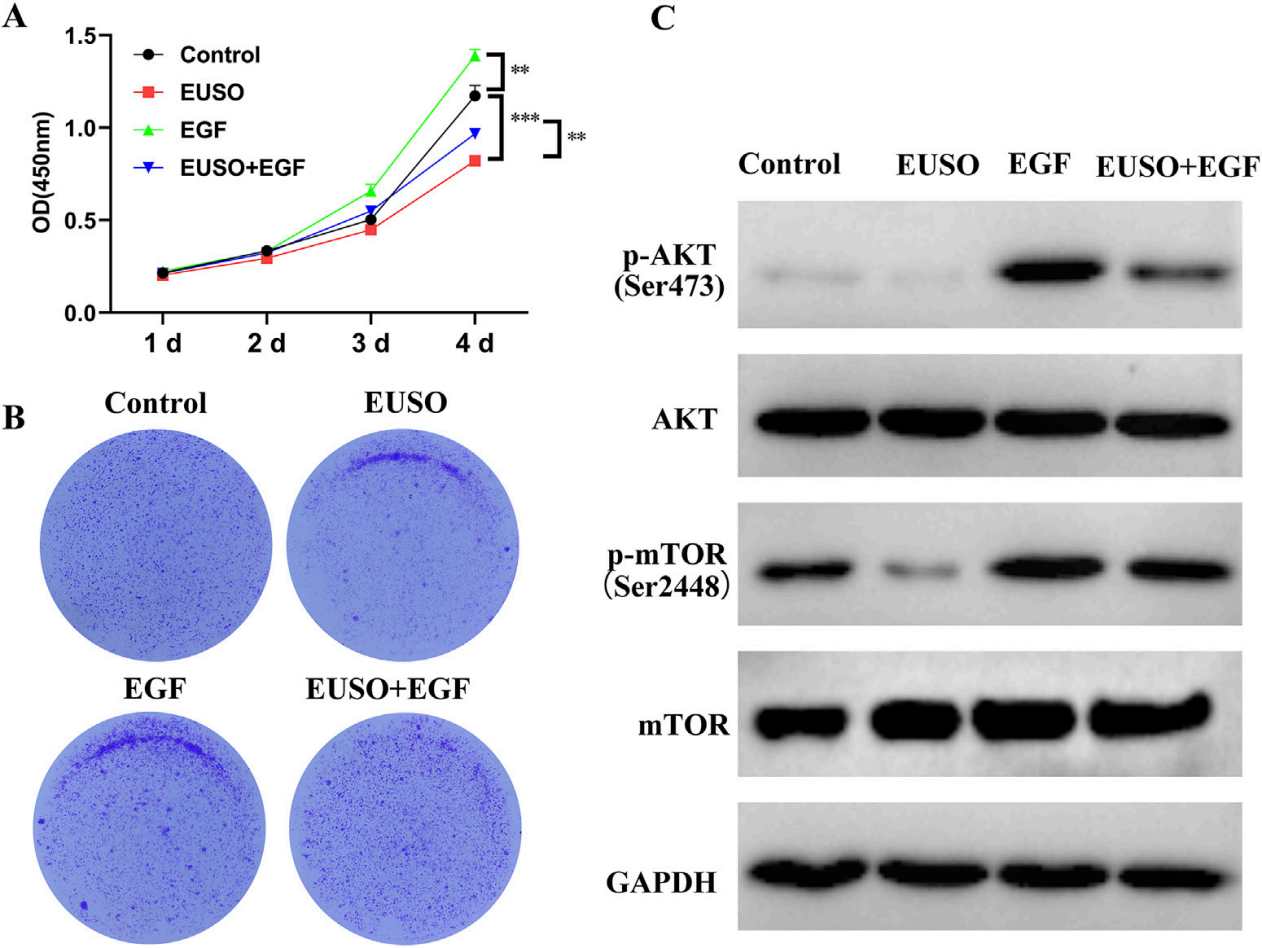
EUSO inhibits tumor malignant transformation through the PI3K-AKT-mTOR pathway. **(A)** CCK8 assays revealed that overexpression of AKT could rescue the inhibitory effects of EUSO on cell proliferation, **(B)** Colony formation assays revealed that overexpression of AKT could rescue the inhibitory effects of EUSO on cloney formation, **(C)** Western blot (WB) analysis demonstrated that overexpression of AKT also reversed the reduction of p-AKT (Ser473) and p-mTOR (Ser2448) protein levels induced by EUSO.

CCK8 and colony formation assays revealed that overexpression of AKT could rescue the inhibitory effects of EUSO on cell proliferation and clonogenicity (Figures 5A,B). Western blot (WB) analysis demonstrated that overexpression of AKT also reversed the reduction of p-AKT (Ser473) and p-mTOR (Ser2448) protein levels induced by EUSO (Figure 5C). These findings suggest that EUSO suppresses the malignant phenotype of tumor cells through the PI3K-AKT-mTOR pathway.

### 3.6 EUSO inhibits the growth of xenografts *in vivo*

**FIGURE 6:**
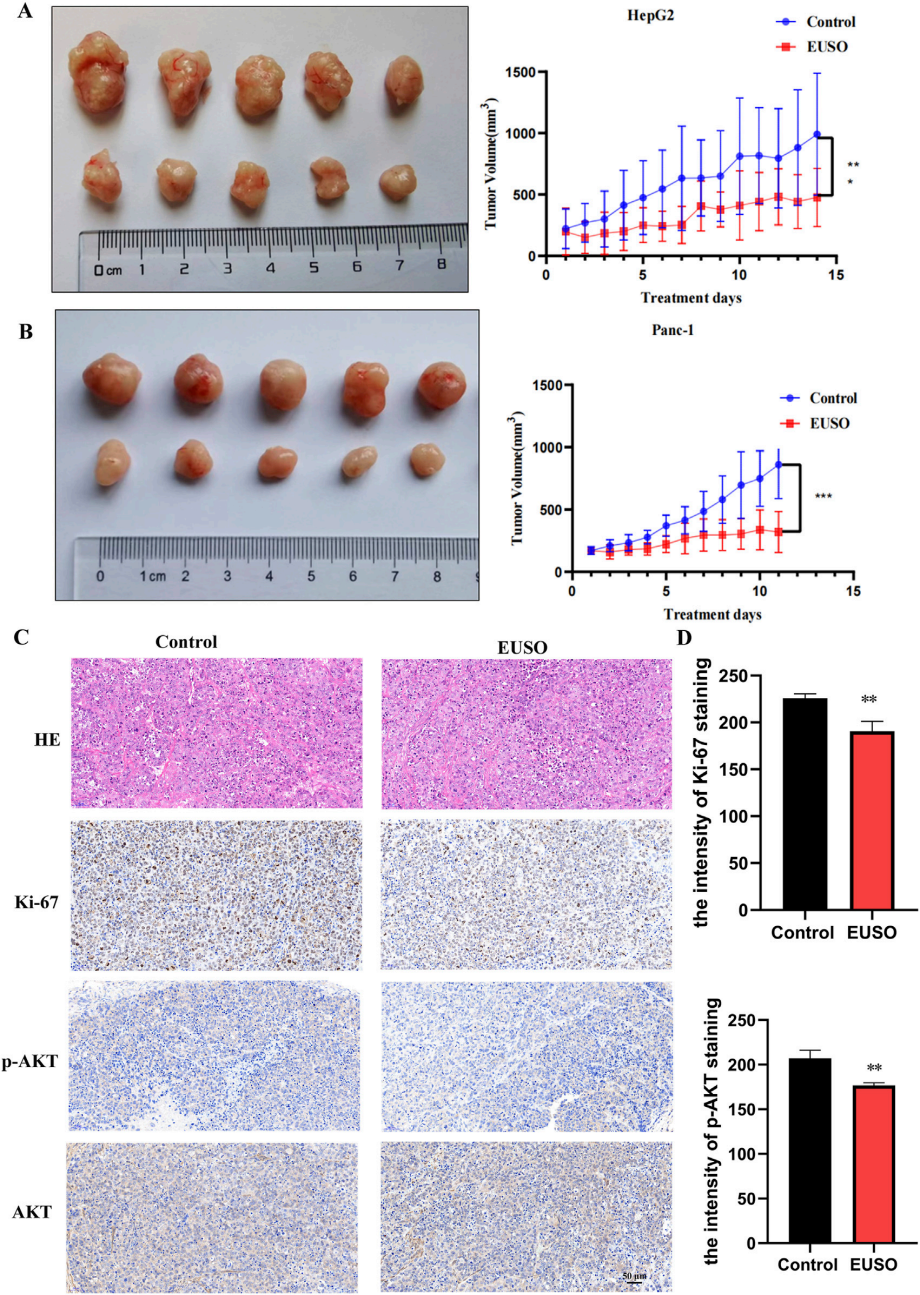
EUSO *in vivo* inhibits the growth of transplanted tumors in mice. **(A)** The tumor volume formed by Panc1 cells was significantly suppressed in mice treated with EUSO compared to controls, **(B)** The tumor volume formed by HepG2 cells was significantly suppressed in mice treated with EUSO compared to controls, **(C,D)** Ki-67 and p-AKT staining showed that the proportion of positive tumor tissue and staining intensity in the EUSO-treated group were significantly weaker than those in the control group. The difference was statistically significant. **p < 0.01, ***p < 0.001.

HepG2 and Panc-1 cells were subcutaneously injected into nude mice to establish tumor xenografts. Once tumors were successfully established, the mice were randomly divided into a control group and an experimental group. The experimental group received EUSO treatment via oral gavage for 14 days, while the control group was administered an equivalent volume of water. After 14 days, the animals were euthanized, and tumor tissues were carefully dissected for analysis. Tumor volumes were significantly reduced in the EUSO-treated group compared to the controls (Figures 6A,B). Immunohistochemical staining for Ki-67 and phosphorylated AKT (p-AKT, Ser473) showed a marked decrease in both the proportion of positive tumor cells and staining intensity in the EUSO-treated group relative to the control group (Figures 6C,D), indicating that EUSO effectively inhibits tumor cell proliferation *in vivo*.

## 4 Discussion

Natural medicines and their bioactive components represent a promising frontier for developing therapeutic agents against cancer, offering significant potential to improve patients’ quality of life (Zhao et al., 2023; Zhang et al., 2024). Notably, more than 50% of recently developed anti-tumor drugs originate from natural sources and combat cancer through various mechanisms, including proliferation inhibition, apoptosis induction, angiogenesis suppression, and immune system enhancement (Beilankouhi et al., 2023; Kulbay et al., 2022; Liu et al., 2021; Hsu et al., 2021). Among these resources, medicinal-food homologous plants are particularly promising due to their multi-component nature and diverse biological activities, making them valuable for pharmaceutical development, functional foods, and nutraceuticals.

Eucommia ulmoides, a dual-purpose plant recognized in both traditional medicine and dietary practice, contains a key bioactive component in its seeds-EUSO (Li et al., 2022; Li et al., 2024; Mo et al., 2022; Tang et al., 2023). Previous studies have demonstrated EUSO’s broad therapeutic properties, including anti-osteoporotic, anti-inflammatory, antimicrobial, hypotensive, lipid-lowering, immunomodulatory, and antioxidant effects (Shi et al., 2022; Meng et al., 2022). In this study reveals that EUSO exhibits notable specificity against digestive system cancers by suppressing tumor cell proliferation, clonogenicity, migration, and tumorigenesis. Mechanistically, EUSO induces apoptosis through significant downregulation of the anti-apoptotic protein Bcl-2 and upregulation of the apoptotic effector cleaved caspase-3. These properties suggest that EUSO has potential as a basis for developing tumor adjuvant drugs.

The PI3K-AKT-mTOR signaling pathway is essential for maintaining normal cellular homeostasis and has also emerged as a critical oncogenic driver in tumorigenesis and cancer progression (Revathidevi and Munirajan, 2019; Khezri et al., 2024). PI3K-AKT-mTOR pathway have aberrant activation in more than 29% of patients with multiple types of malignancies and regulate key tumor processes including angiogenesis, cell proliferation, metabolism, migration and apoptosis (Hoxhaj and Manning, 2020; Yang et al., 2019). In our study, our findings demonstrate that EUSO exerts its anti-tumor effects in digestive system cancers by suppressing the PI3K-AKT-mTOR cascade, thereby disrupting these key cancer-promoting mechanisms. This mechanistic insight positions EUSO as a promising modulator of this therapeutically pivotal pathway in digestive system cancer cells malignancies.

## 5 Conclusion

In conclusion, we demonstrated that EUSO effectively inhibits digestive system cancer cells proliferation, migration, and tumorigenesis while inducing apoptosis through the reduction of the PI3K-AKT-mTOR signaling pathway. These findings provide a theoretical basis for the use of EUSO as an adjuvant food in tumor therapy.

## Data Availability

The data that support the findings of our study are available on request from the corresponding authors.
